# Gut-immunity-joint axis: a new therapeutic target for gouty arthritis

**DOI:** 10.3389/fphar.2024.1353615

**Published:** 2024-02-23

**Authors:** Pei Chen, Zhiqiang Luo, Chengyin Lu, Gonghui Jian, Xinyu Qi, Hui Xiong

**Affiliations:** ^1^ Hunan University of Chinese Medicine, Changsha, Hunan, China; ^2^ The Second Hospital of Hunan University of Chinese Medicine, Changsha, Hunan, China; ^3^ The First Hospital of Hunan University Chinese Medicine, Changsha, Hunan, China; ^4^ College of Integrative Chinese and Western Medicine, Hunan University of Chinese Medicine, Changsha, Hunan, China

**Keywords:** gouty arthritis, gut microbes, immune response, macrophage, inflammation, pain mechanism

## Abstract

Gouty arthritis (GA) is an inflammatory disease characterized by pain. The primary goal of current treatment strategies during GA flares remains the reduction of inflammation and pain. Research suggests that the gut microbiota and microbial metabolites contribute to the modulation of the inflammatory mechanism associated with GA, particularly through their effect on macrophage polarization. The increasing understanding of the gut-joint axis emphasizes the importance of this interaction. The primary objective of this review is to summarize existing research on the gut-immune-joint axis in GA, aiming to enhance understanding of the intricate processes and pathogenic pathways associated with pain and inflammation in GA, as documented in the published literature. The refined comprehension of the gut-joint axis may potentially contribute to the future development of analgesic drugs targeting gut microbes for GA.

## 1 Introduction

Gouty arthritis (GA) is a crystalline arthritis caused by the deposition of monosodium urate (MSU) (Dalbeth et al., 2021). It is directly related to hyperuricemia (HUA), which results from impaired purine metabolism, and is recognised as one of the most distressing conditions for humans (Kuo et al., 2015). During GA flares, patients experience intense, knife-like joint pain that lasts for days, significantly affects their quality of life (Richette et al., 2020). In recent years, the prevalence of HUA and GA has steadily increased with improvements in living standards and dietary habits (Zhou M. et al., 2019). HUA and GA have a significant global impact, with an estimated prevalence exceeding 1 billion (Zhang M. et al., 2021). The incidence and prevalence of GA increases with age (Dalbeth et al., 2021). GA has emerged as a critical global public health issue, with the alleviation of GA pain forming the core of treatment (Stamp and Dalbeth, 2022).

The gut serves as a pivotal organ for digestion and absorption, as well as the largest immune organ (Wei et al., 2022). With advancements in our understanding of gut microbes, studies confirm their crucial roles in both acute and chronic pain disorders (Wang Y. et al., 2022). While there is no direct physical connection between the gut and joints, epidemiological studies suggest a possible relationship between the gut health and arthritis (Zundler et al., 2023). Inflammatory arthropathies are often preceded by subclinical intestinal inflammation or intestinal barrier disorders! Changes in gut microbes and intestinal barrier dysfunction also contribute to arthropathies (Tajik et al., 2020; Sun et al., 2023). Anti-acute gout arthritis (AGA) analgesic drugs such as nonsteroidal anti-inflammatory drugs (NSAIDs), colchicine(COL), glucocorticoids, and botanical drugs exert anti-inflammatory and analgesic effects by acting on inflammatory pathways once they are absorbed into the systemic circulation (Shi et al., 2020; Han R. et al., 2021; Tong et al., 2022). Gut microbes also play a key role in the pharmacological effects of these drugs. Pain is the primary characteristic of inflammation (Fitzcharles et al., 2021). Macrophages are critical cells in the inflammatory machinery and their inflammatory-pain channels are significant in activating sensory neurons associated with GA pain (Lan et al., 2021; Zaninelli et al., 2022). Therefore, this paper reviews the influence of gut microbiota on macrophage-mediated inflammatory and pain mechanisms in GA from the perspective of the gut-immune-joint axis. Its aim is to provide insights for GA diagnosis and treatment, as well as a reference for the development of new anti-GA analgesic drugs.

## 2 Macrophages contribute to the inflammatory pain mechanisms of GA

**FIGURE 1:**
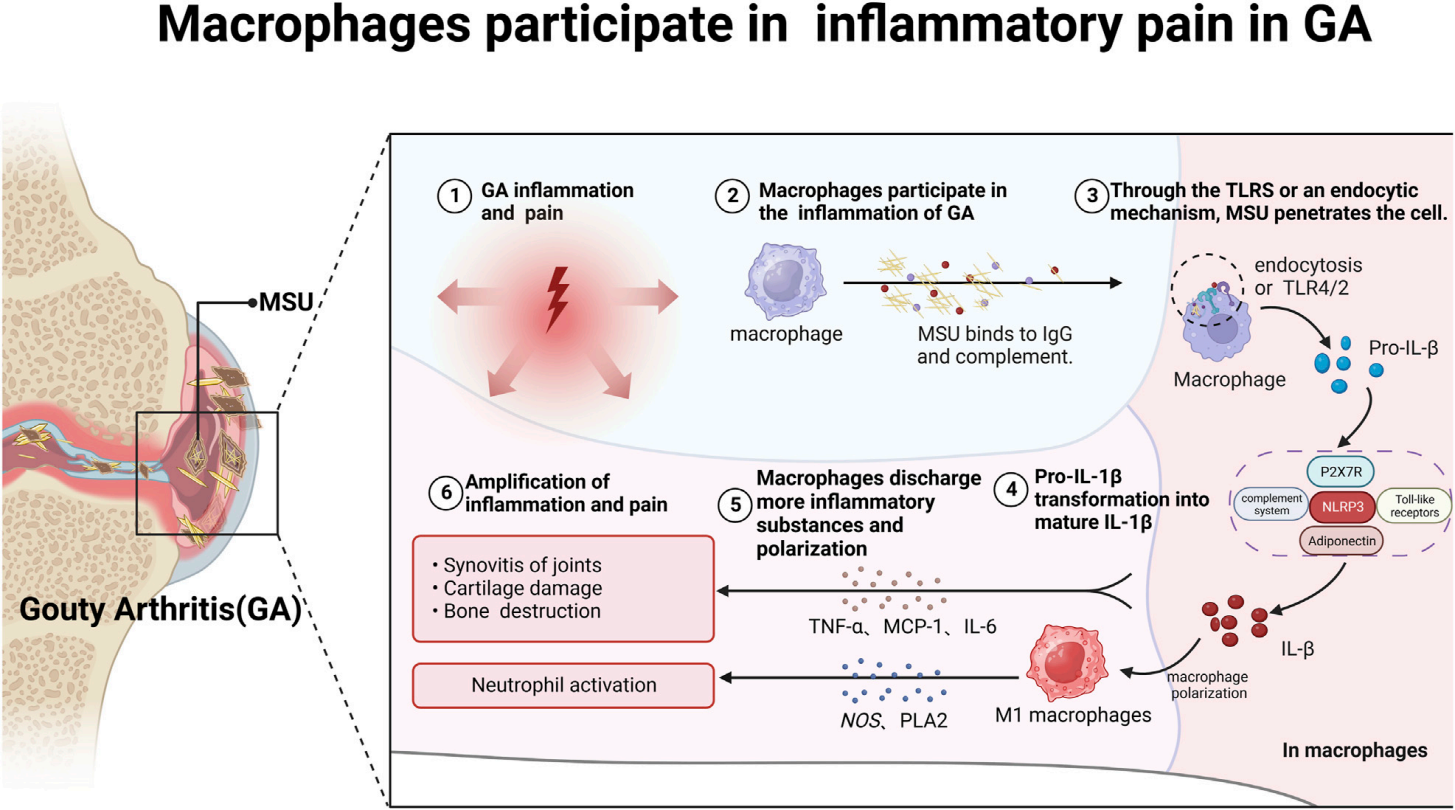
Macrophages participate in mediating inflammatory pain in GA. After binding to complement and IgG, MSU crystals enter macrophages via TLRs or cytosolic pathways, triggering immune and inflammatory responses and storing a large amount of pro-IL-1β. Subsequently, pro-IL-1β is delivered to the NLRP3/ASC/Caspase signaling pathway, promoting the conversion of pro-IL-1β into mature IL-1β and the secretion of other inflammatory factors such as TNF-α, MCP-1 and IL-6. On the one hand, IL-1β can promote macrophage polarization into M1 macrophages; on the other hand, it can form a positive feedback loop, further exacerbating GA pain.

The macrophage-mediated inflammatory response plays a pivotal role in GA pain pathogenesis (Dalbeth et al., 2021). MSU crystals are recognized by macrophages via Toll-like receptors (TLRs) and ingested, leading to activation of the NLRP3 inflammasome and cleavage of pro-interleukin 1β (pro-IL-1β) into mature IL-1β (Zhou F. et al., 2019; Cabău et al., 2020; Cavalli et al., 2021). IL-1β is a potent pro-inflammatory cytokine central to GA inflammation and pain, enhances excitatory neuron transmission and inhibits inhibitory synapses, thereby amplifying nociceptive signaling to the central nervous system (Liu T.-W. et al., 2022). It enhances excitatory neuron transmission and inhibits inhibitory synapses, amplifying nociceptive signaling to the central nervous system (Mailhot et al., 2020). Additionally, activated macrophages also secrete hyperalgesic cytokines such as TNF-α, MCP-1, and IL-6, which promote polarization into pro-inflammatory M1 macrophages (Ren et al., 2021; Zhao J. et al., 2022; Nieradko-Iwanicka, 2022). M1 macrophages release reactive oxygen and nitrogen species, cyclooxygenase-2, and phospholipase A2, perpetuating inflammation and sensitizing TRPA1 pain channels (Yang and Xing, 2021) (Figure 1).

By inhibiting macrophage apoptosis, this establishes a self-sustaining cycle of inflammation and pain in GA (Zhao et al., 2022d). In conclusion, macrophages play an indispensable role in the pathogenesis of pain and inflammation in GA through activation of the Nod-like receptor family pyrin domain-containing protein 3 (NLRP3) inflammasome and production of pro-inflammatory cytokines. Strategically targeting the activation pathways of macrophages presents a promising therapeutic approach for mitigating GA.

It is noteworthy that macrophages persist throughout the development, progression, and regression of GA by differentiating into pro-inflammatory M1 and anti-inflammatory M2 macrophage phenotypes (Zhao et al., 2022c). Recent studies have challenged classical the M1/M2 dichotomy, proposing instead that macrophages can be classified into subsets based on the expression of TREM2 and FOLR2 genes (Nalio Ramos et al., 2022). The gut microbiota and their metabolites significantly influence the regulation of macrophage activation states and function. Dysbiosis gut bacteria-derived Lipopolysaccharide (LPS) can bind to TLRs, inducing pro-inflammatory activation. Conversely, short-chain fatty acids (SCFAs) produced by commensal microbes exhibit anti-inflammatory effects on macrophages. A cohort of twenty patients diagnosed with AGA participated in this study. Blood and fecal samples were systematically collected over a 3-day duration, capturing patients at varying stages of acute manifestation and subsequent recovery. The analysis of the stool microbiome employed 16S rRNA sequencing, while SCFAs were quantified using gas chromatography-mass spectrometry. The investigation aimed to discern disparities in both the gut microbiome and SCFAs between the acute and recovery phases in individuals afflicted with GA. The findings revealed noteworthy alterations in bacterial composition that facilitated the production of SCFAs, particularly acetate, following the implemented treatment(Park and Lee, 2022).Targeting the gut microbiota-macrophage axis is representing a promising approach to reducing inflammation in GA. Clarifying the specific gut microbial metabolites and signaling pathways that influencing macrophage polarization will enable the development of novel microbiome-based therapies for managing GA pain and inflammation.

## 3 Exploring macrophage extracellular traps in the pathogenesis of GA

Macrophage Extracellular Traps (METs) are specialized structures formed by macrophages, responding to stimuli like infections. METs, which consist of released chromatin and proteins, function to trap and eliminate pathogens. Their metabolites activate immune responses, making them crucial for bolstering the immune system’s defense mechanisms. The identification of METs has recently provided new insights into the pathogenesis of inflammatory diseases, including GA (Weng et al., 2024; Weng et al., 2024). In the context of rheumatoid arthritis (RA), METs can activate fibroblast-like synoviocytes through TLR signaling, thus perpetuating the inflammatory response (El Shikh et al., 2019). Previous studies on extracellular traps in GA have mainly concentrated on neutrophil extracellular traps (NETs) (Apel et al., 2018), the involvement of METs in this context remains elusive.

Considering the pivotal role that macrophages play in inflammation associated with GA, an exhaustive examination of the formation and function of NETs may elucidate previously overlooked mechanisms linking gut dysbiosis to joint inflammation. In a DSS-induced mouse model of colitis, the expression profiles of pro-inflammatory cytokines and chemokines were evaluated after oral administration of *butyrate* by researchers. The migration and release of NETs were also examined through the Transwell model and immunofluorescence using qRT-PCR and ELISA, respectively. The study indicates that oral administration of *butyrate* can effectively reduce mucosal inflammation in DSS-induced colitis in mice. This positive outcome was achieved by suppressing neutrophil-associated immune responses, including pro-inflammatory mediators and NET formation (Li et al., 2021). Metabolites within NETs may subsequently activate synovial cells and sensory neurons, contributing to the onset and progression of pain and inflammation (Tansley et al., 2022).

A better understanding of NETs as inflammatory triggers in GA pathology could reveal new microbiome-based therapies to reduce macrophage activation and limit NET release. Improved comprehension of NET signaling in GA will undoubtedly provide valuable insights into the various roles of macrophages in promoting joint inflammation caused by gut dysbiosis.

## 4 Gut microbes intervene in the “inflammation-pain” mechanism of GA via the macrophages

### 4.1 TLRs overview: molecular mechanisms in the pathogenesis of GA

TLRs are membrane receptors that are critically involved in immune responses and contribute to shaping the microenvironment of inflammation (Arleevskaya et al., 2020). There are thirteen TLRs known to be recognized in mammals, namely, TLR1 to TLR13 (Lind et al., 2022). Current investigations into TLRs associated with GA are mainly focused on TLR2 and TLR4, while TLR1, TLR3 and TLR5 have also been reported in a few studies (Joosten et al., 2016). The ligands for TLRs identified so far include LPS, F protein, sodium urate, paclitaxel, heat shock protein 60, and fibronectin (Li and Wu, 2021). Upon recognition of their ligands, TLRs initiate the inflammatory response in GA by activating downstream signal transduction molecules. This activation leads to the stimulation of the TLR signaling pathway, culminating in the release of inflammatory factors such as IL-1β and TNF-α (Elsaid et al., 2023).

**FIGURE 2:**
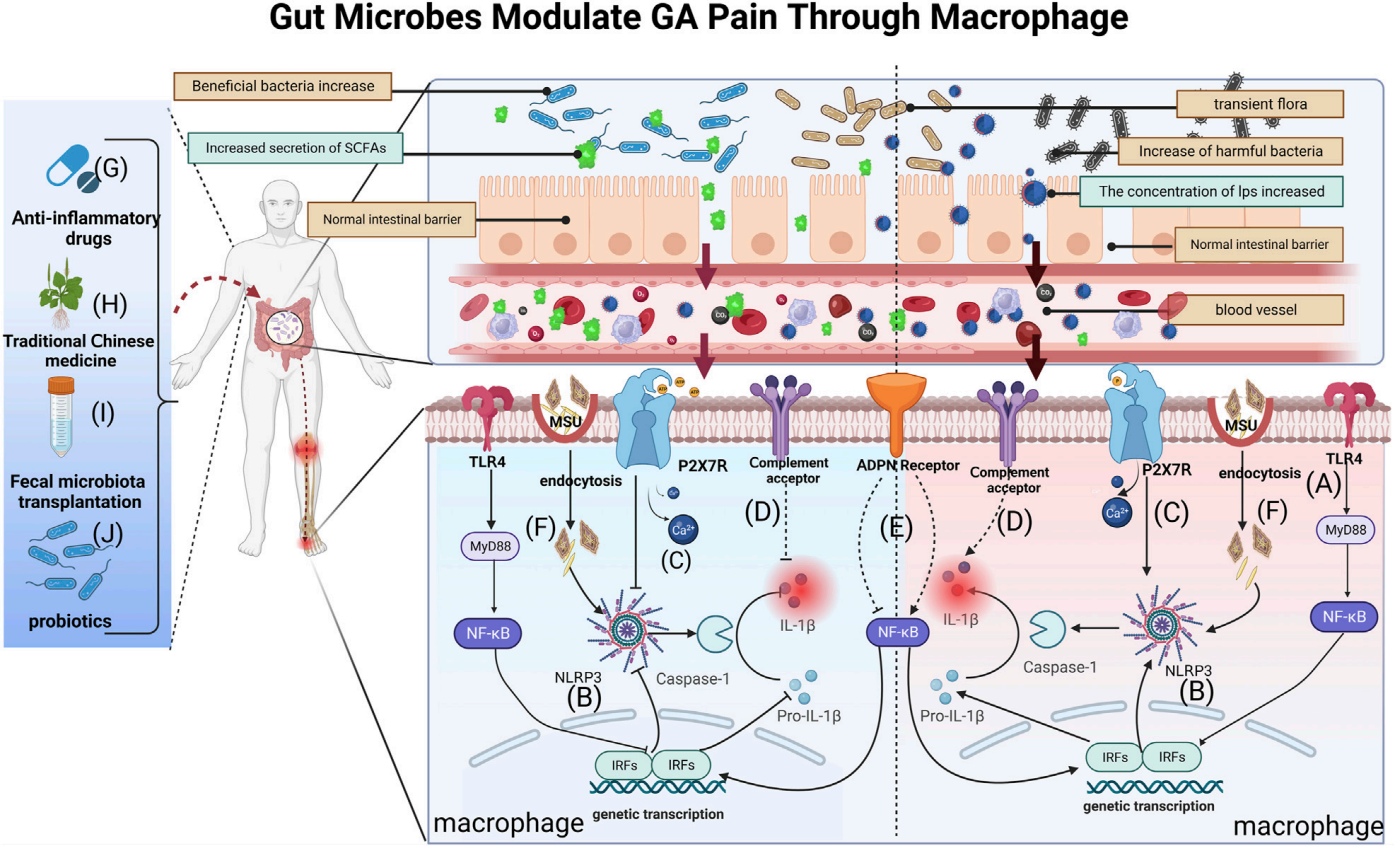
Gut bacteria and their metabolites can reach blood vessels, exert local effects in joints, modify macrophage-mediated inflammation and influence GA pain through breaches in the intestinal barrier. **(A)** LPS activates TLR4 receptors on macrophage membranes and induces IL-1β secretion via the TLR4/NK-κB pathway, causing GA inflammation and pain; **(B)** SCFAs and LPS affect GA pain by activating or inhibiting the NLRP3 pathway in macrophages, regulating IL-1β release; **(C)** P2X7R pathway activation following dysbiosis further regulates inflammatory signaling (e.g., the NLRP3 pathway), promoting mature IL-1β secretion and amplifying pain; **(D)** Complement activation by gut microbes may modulate GA pain by influencing metabolite levels and IL-1β secretion; **(E)** Gut microbiota may influence the NF-κB pathway by regulating ADPN expression in macrophages, affecting IL-1β maturation and secretion; **(F)** After MSU enters into macrophages via cytosolic mode, it promotes the conversion of Pro-IL-1β to mature IL-1β through NLRP3/ASC/Caspase-1-mediated inflammatory events.**(G)** Anti-GA drugs could affect macrophage-mediated GA pain mechanisms by influencing gut microbiota and metabolite concentrations; **(H)** TCM modulates macrophage immunity and inflammation alleviating GA pain through gut microbes and metabolites; **(I)** FMT is likely to treat GA pain by affecting BUA levels and inhibiting M1 macrophage polarization via gut microbes; **(J)** Probiotics reduce GA pain by restoring the intestinal barrier and inhibiting NLRP3 in macrophages, reducing inflammatory factors like IL-1β secretion.

Gut microbe cell-wall components are a significant trigger for inflammation development (Tong et al., 2022). Gram-positive cell wall components Lipoprotein and peptidoglycan can stimulate TLR2 (Gowing et al., 2017), while LPS activates TLR2 and TLR4, respectively (Francisco et al., 2021). These metabolites collectively induce local inflammation and pain hypersensitivity, promoting the synthesis and secretion of inflammatory factors, including IL-1β, TNF-α, and IL-6, through the intramembrane TLR inflammatory signaling pathway. This activation, in turn, triggers corresponding receptors in sensory neurons. In the gut-kidney axis, an examination of the C57BL/6J hyperuricemia mouse model before and after *folic acid* treatment showed that *folic acid* reversed the changes in intestinal microecology induced by hyperuricemia. These alterations encompassed changes in the structure and species composition of the gut microbiota, along with modifications in metabolites, particularly SCFAs (Wang P. et al., 2022) (Figure 2A). Alterations in gut microbial composition can compromise the intestinal barrier, facilitating the entry of LPS into the bloodstream. Subsequently, LPS activates the TLR4/NF-κB inflammatory signaling pathway, leading to the upregulation of various inflammatory factors. Ultimately, this cascade results in nociceptive hypersensitivity and GA flares (Liu Z.-Q. et al., 2022). A cohort of 15 *Anser cygnoides* goslings, displaying typical symptoms of visceral gout, was meticulously selected and compared with a control group of 15 healthy goslings. Microbiome signatures in the cecum chyme of both groups were characterized through 16S sequencing. Additionally, assessments were conducted to evaluate changes in intestinal permeability, serum LPS levels, and the TLR-mediated inflammatory response. The investigation revealed that goslings with gout exhibited gut dysbiosis resulting from intricate interactions among diverse gut bacteria. The proliferation of the pathogenic genus Proteobacteria was identified as a key factor in this dysbiosis. Furthermore, the gout-afflicted group demonstrated an elevation in systemic LPS concentration, activating the LPS/TLR4/MyD88 inflammatory signaling pathway (Xi et al., 2019).

The researchers conducted a systematic exploration of relevant literature from major databases. They found increased levels of *Bifidobacterium, Lactobacillus, Bacteroidetes,* and *Prevotella*. In contrast, they identified a decrease in *Aspergillus* abundance and altered thick-walled *Bacteroidetes/Anthrobacteroidetes* ratios, which led to subsequent changes in their metabolites. Alterations in the gut microbiota were induced by SCFAs and LPSs, resulting in the downregulation of the TLR4/NF-κB inflammatory signaling pathway and the inhibition of xanthine oxidase activity (Liu Z.-Q. et al., 2022).

### 4.2 Role of NLRP3 inflammasome in the mechanism of GA

Inflammasomes, intricate protein complexes, represent crucial components within the immune system (Wang et al., 2023). Inflammasomes, pivotal in orchestrating the inflammatory response, play a significant role in generating inflammatory cytokines that subsequently influence sensory neuron function (Rathinam and Fitzgerald, 2016). In recent years, inflammasomes have received significant scholarly attention due to their involvement in pain signaling (Sreejit et al., 2022). When MSU is recognized by the model receptor, the receptor will promote the synthesis of the NLRP3 inflammasome and the release of mature IL-1β, which will induce the onset of GA flares and pain. In patients with AGA, researchers synovial macrophages were isolated and demonstrated a notable reduction in MSU crystal-induced GA through the genetic ablation or pharmacologic inhibition of NLRP3 function (Lan et al., 2021). A clinical trial was conducted on 34 patients diagnosed with monoarticular gout flare confirmed by MSU crystals. The patients were divided into four dosage groups and orally administered dapansutrile, an oral selective NLRP3 inflammasome inhibitor, for 8 days. The primary outcomes evaluated changes in patient-reported pain in the target joint from baseline to days 3 and 7. The study showed a significant decrease in joint pain in all dosage groups, with the 300 mg/day group having the most significant effect, and a significant decrease in joint pain in all dosage groups, with the 300 mg/day group having the most significant effect. Dapansutrile was found to be safe and effective in relieving AGA pain (Klück et al., 2020). It is likely that MSU activates the NLRP3 inflammasome in macrophages through the reactive oxygen pathway, tissue protease pathway, and K+ efflux pathway. However, a central question in GA research persists: What specific pathway do MSU crystals follow to activate the NLRP3 inflammasome, leading to IL-1β release? This question remains crucial for comprehending the underlying mechanisms in the future.

Gut microbes and their metabolites are known to induce GA pain through the NLRP3 inflammasome signaling pathway (Lin et al., 2020; Wang et al., 2023). Studies have focused on two pathways (Figure 2B): LPS participates in the pathway and SCFAs are involved in the pathway(Wen et al., 2020; Sun et al., 2022). Among them, SCFAs, also known as volatile fatty acids (VFAs), mainly include *acetic acid*, *propionic acid*, *isobutyric acid*, *butyric acid*, *isovaleric acid*, and *valeric acid* (Wu Y.-L. et al., 2022; Yao et al., 2022). These molecules are able to influence the onset of pain through the regulation of macrophage polarization and inflammation (Leong et al., 2022; Liu et al., 2023). One study showed that an increase in beneficial bacteria such as *Rikenella* and *Alistipes*, coupled with a reduction in potentially harmful bacteria such as *Escherichia-Shigella* and a decrease in LPS-bearing Gram-negative bacteria in an autoimmune hepatitis (AIH) mouse model, could inhibit the TLR4/NF-κB and NLRP3/Caspase-1 pathways in AIH (Kang et al., 2023). Therefore, targeting gut microbes and their metabolites to modulate macrophage polarization and, consequently, mitigate GA immune inflammation holds promise as a clinical therapeutic strategy.

### 4.3 Inflammatory signaling cascades: connecting P2X7 receptor to GA development

IL-1β is an essential inflammatory transmitter in GA flares (Spel and Martinon, 2020). Recently, a study reported that there was no significant difference in the elevated IL-1β levels between patients with GA and patients with HUA when MSU was stimulated alone. But when co-stimulated with MSU and adenosine triphosphate (ATP), levels of IL-1β were found to be significantly higher in patients with GA than patients with HUA, which suggests that ATP is a second pathogenic signal for GA in addition to MSU (Wu L. et al., 2022). ATP is a high-energy phosphate metabolite that is the most direct source of energy in living organisms (Genetzakis et al., 2022). Dramatic fluctuations in ATP are present in triggers of GA flares, such as alcohol abuse and overeating (Richette et al., 2020). The purinergic receptor P2X, ligand-gated ion channel 7 (P2X7), is a critical factor in the processing and release of IL-1β, which plays an important role in GA flares (Wu L. et al., 2022; Su et al., 2019). P2X7 expression is mainly associated with immune cells such as macrophages (Genetzakis et al., 2022). It has unique biological characteristics, including involvement in the processing and release of various inflammatory factors such as IL-1β, IL-18 and TNF-α (Li M.-Y. et al., 2023). It was found that infiltration and secretion of pro-inflammatory factors were quantified in fresh or cultured mouse peritoneal macrophages treated with COL *in vitro* or *in vivo*. The results showed that mouse peritoneal macrophages in the presence of COL exhibited less ATP-induced permeabilisation to ethidium bromide and less reactive oxygen species (ROS) formation, nitric oxide (NO) and IL-1β release. This finding confirms the significant role of ATP and P2X7R in the pathogenesis of GA flares (Marques-da-Silva et al., 2011). In addition, ATP is an important signal for GA, and the function and status of P2X7R may be an essential factor in GA flares (Li M.-Y. et al., 2023). The mechanism is that P2X7R activation causes Ca2^+^ inward flow, which synergistically activates NLRP3 with MSU, resulting in the secretion of large amounts of IL-1β by macrophages (Li X. et al., 2023). Thus, P2X7R, as a pro-inflammatory receptor, plays a critical role in the development of inflammation and pain during GA flares.

The gut is an important organ for digestion and absorption, which are closely related to energy metabolism (de Vos et al., 2022). As research on the gut-disease axis grows, researchers are also paying more attention to the interaction of gut microbes with purinergic signaling pathways and the biological effects they play in the evolution of disease (Li M. et al., 2023). In a therapeutic study, P2X7R^+/+^ mice were treated with the P2X7R selective inhibitor A740003.Video endoscopy and endoluminal ultrasound biomicroscopy revealed histological changes in colonic tissue.Analyses showed increased accumulation of immune cells, increased proinflammatory cytokine production and upregulated NLRP3 and NLRP12 genes in P2X7R^+/+^ mice, which were alleviated by P2X7R inhibition. Microbial alterations in P2X7R^−/−^ and P2X7R+/+-induced mice were partially reversed by A740003, suggesting that the interaction between P2X7R and dysbiotic microbiota signals triggers intracellular pathways, inflammasome activation and exacerbates inflammation (Bernardazzi et al., 2022) (Figure 2C). In a murine model of alcoholic liver disease (ALD), carnosic acid (CA) mitigates ethanol-triggered lipid accumulation in AML12 cells and diminishes IL-1β release by suppressing the P2X7R-NLRP3 pathway in murine peritoneal macrophages (MPM) (Zuo et al., 2023). Thus, it would be a promising research field for the development of GA-analgesic drugs.

### 4.4 Targeting the complement activation pathway: therapeutic implications in GA treatment

The complement system, recognized as the activating pathway of complements, constitutes a significant element within the innate immune system (Rahal et al., 2021). Initially, the prevailing belief was that the complement system’s primary function lay in recognizing and eliminating pathogens exclusively through the stimulation of phagocytosis by immune cells (Killick et al., 2018). However, with the revelation of immunomodulatory functions extending beyond pathogen elimination, complement proteins have been shown to play a key role in mediating inflammation and regulating cellular immunity (An et al., 2014). Dysregulation of the complement system has been found to be closely associated with the pathogenesis and clinical manifestations of GA. Activation of the complement system via the classical, alternative, and lectin pathways triggers an inflammatory response mediated by immune cells, resulting in GA inflammatory damage and pain. The surface of MSU crystals from patients with GA is encapsulated with complement, which are able to activate both classical and alternative pathways of complement activation (Khameneh et al., 2017). MSU-associated complement mediates inflammatory responses in GA by stimulating immune cells to produce IL-1β. In contrast, inhibition of complement system activation can decrease the inflammatory response of GA (West and Kemper, 2023). As reported by Ling-Ling An *et al.*, MSU crystal-induced proinflammatory cytokines/chemokines in human whole blood is predominantly regulated by C5a through its interaction with C5a receptor. C5a induces pro-IL-1β and IL-1β production in human primary monocytes, and potentiates MSU or cholesterol crystals in IL-1β production (An et al., 2014). Hence, the activation of the complement system enhances immune responses and is acknowledged as a crucial factor in various inflammatory diseases, including GA. Despite studies confirming MSU’s ability to activate the classical pathway of complement activation, the precise mechanism remains unclear.

The gut microbiota has been acknowledged to be vital in the development of metabolic-immune illnesses, functioning as crucial metabolic and inflammatory regulators (Lai et al., 2022). Moreover, an increasing body of literature highlights the significance of the gut microbiota in modulating the complement system. Research indicates that gut microbes exert influence on disease progression by manipulating gene expression, altering metabolite levels, activating the complement system, and impacting local and systemic immune responses (Figure 2D). A study investigated the influence of C4B gene number on gut microbiota and *in vitro* serum complement activation in patients with paediatric inflammatory bowel disease (PIBD). Using genomic reverse transcription-polymerase chain reaction (RT-PCR), the researchers found a positive correlation between C4B gene number and inflammation in PIBD. Elevated C4B gene copies may exacerbate dysbiosis in inflammatory bowel disease by increasing complement reactivity to the microbiota (Nissilä et al., 2017). Using a rat thoracotomy pain model, the researchers assessed pain behaviour and the expression of molecular markers. Employing molecular profiling techniques including RT-PCR, Western blot, immunofluorescence and single cell RNA sequencing, the study showed that downregulation of C3aR inhibited A1 astrocyte activation, leading to reduced expression of C3aR, C3 and GFAP.This led to alleviation of mechanical withdrawal thresholds and chronic pain (Zhu et al., 2023). Despite studies demonstrating the relevance of the gut microbiota to the complement system, scant research has investigated whether gut microbes and their metabolites influence autoimmune diseases such as GA through effects on the complement system.

### 4.5 Exploring the future of adiponectin in GA: advancing understanding and intervention strategies

Adiponectin (ADPN) is one of the most abundant adipokines in plasma (Fiaschi, 2019). It is abundantly secreted and expressed in adipose tissue and secreted in skeletal muscle cells, cardiac myocytes, endothelial cells, and various other cell types (Fang and Judd, 2018). Currently, ADPN is currently considered a potential pro-inflammatory mediator that acts primarily through its receptor (Łączna et al., 2022). The abnormal expression of ADPN and its receptor genes in GA patients suggests that ADPN may contribute to IL-1β-mediated inflammation through receptor signaling. In a study of 90 consecutive patients in a crystal arthritis unit, increased levels of interleukin-18 (IL-18), soluble interleukin-6 receptor (sIL-6R), regulated upon activation, normal T cell expressed and secreted (RANTES), leptin and ADPN were found in patients with intermittent gout compared with their controls (Diaz-Torne et al., 2021). However, it has also been reported that ADPN plays an anti-inflammatory role in GA and is negatively associated with BUA levels. In a study comprising 258 male gout patients and 111 males undergoing annual check-ups, elevated levels of leptin and plasminogen activator inhibitor-1, coupled with reduced ADPN and ADPN/leptin ratio, were noted in patients with gout (*p* < 0.05, respectively). These findings imply a significant role for ADPN in the pathogenesis of gout (Inokuchi et al., 2010). Interestingly, Ruyi Cong *et al.* evaluated whether there is a causal relationship between specific ADPN and the development of HUA and GA through a two-sample Mendelian randomization study. However, the results found that ADPN may not play a causal role in the development of HUA and GA (Cong et al., 2022). Nevertheless, multiple findings support the notion that lipocalin may reduce BUA levels in GA patients, although the exact mechanism remains unclear. Further investigation is imperative to ascertain the correlation between ADPN and inflammatory markers in GA, elucidating the regulatory mechanism as either positive or negative.

Presently, there is a paucity of research investigating the impact of ADPN on GA from the perspective of gut microbiota, given the unclear mechanisms underlying ADPN-induced GA inflammation (Cong et al., 2022). Valuable insights can be gleaned from studies on other inflammatory diseases that have explored the regulatory mechanisms of ADPN in inflammation (Figure 2E). In germ-free (GF) mice, one study discerns the *Lactobacillus NK6* colony, closely associated with *Lactobacillus taiwanensis* strain *BCRC 17755*, as a potential catalyst for inducing FABP4, adipsin, and ADPN expression via TRAF2 and TRAF6 ubiquitination-mediated NF-κB signaling. These discoveries unveil a novel mechanism governing the gut microbiota-mediated expression of FABP4, adipsin, and ADPN in intestinal Paneth cells (Su et al., 2015).

## 5 Effects of conservative GA treatments on inflammatory pain mechanisms via the “gut-immune-joint” axis

### 5.1 Influence of gut microbes on the efficacy at anti-GA analgesic drugs

With advancements in techniques for detecting gut microbes, it has become evident that gut microbes are not only linked to the causative factors of diseases but also influence the effectiveness of their treatment (Ahlawat et al., 2021). In the pathogenesis of GA, macrophages play a crucial role by recognizing and engulfing urate crystals, thereby initiating an inflammatory response. This process triggers the release of inflammatory mediators, resulting in tissue inflammation and the manifestation of pain symptoms (Dalbeth et al., 2021) (Figure 2F). Commonly used in clinical practice during arthritis flares are anti-inflammatory analgesic drugs such as COL, nonsteroidal NSAIDs, and glucocorticoids (Figure 2G). For instance, mice exposed to different doses of COL (0.1, 0.5 and 2.5 mg kg-1 bw per day) for 1 week exhibited severe intestinal injury. The researchers found that COL reduced the expression of pro-inflammatory cytokines and tight junction proteins in ileal and colonic tissues. At 2.5 mg/kg, a profound remodeling of the gut microbiota was observed, potentially increasing intestinal toxicity. Elevated serum levels of diamine oxidase (DAO) and LPS indicated increased intestinal permeability, compromising the intestinal barrier (Shi et al., 2020). NSAIDs like celecoxib and etoricoxib can impact gut microbial growth, subsequently influencing the anti-inflammatory and analgesic effects of these drugs (Hernandez-Sanabria et al., 2020).

An 8-week prednisolone treatment of male C57/Bl6J mice was investigated, revealing loss of bone trabeculae and altered gut microbiota. Microbiota analysis revealed shifts in *Rumatobacteriaceae* and *Bacillariophyceae*, with significant differences in OTUs for *Porphyromonas* and *Clostridiaceae*. Transplantation of faecal material from prednisolone-treated mice into untreated mice induced bone loss, confirming the functional role of the microbiota in glucocorticoid-induced osteoporosis (GIO) (Schepper et al., 2020; van Durme et al., 2014). Although some investigations have explored the effects of glucocorticoids on gut bacteria in other immune disorders, few have specifically addressed their impact in GA (Table 1).

**TABLE 1:** Changes in Gut Microbiota and Outcome Measures Under Different Conservative Treatments for GA.

Categories	Therapeutic schedule	Animal models	Alteration of gut microbiota	Targets and mechanisms	Ref.
anti-GA analge-sic drugs(G)	colchicine	Male Kunming mice with GA	Firmicutes, *Lactobacillus* and Alloprevotella**↑**; Bacteroidetes, *Bacteroides*, Candidatus Saccharimonas, Rikenella, Lachnoclostridium, Lachnospiraceae spp and Clostridiales spp**↓**;	DAO, LPS and intestinal permeability↑, impairing intestinal barrier, inflammatory pathway↓;	Shi et al. (2020)
	febuxostat	AGA patients	Firmicutes, Fusobacteria, Firmicutes, Lachnospiraceae *Clostridium*, Fecalibacterium, Cytophaga, Dorea, Roseburia, Ruminococcaceae *Clostridium*, Clostridiaceae *Clostridium*, Alistipes, Succinispira, Sporobacter, *Campylobacter*, Lachnospira, Robinsoniella, Lactonifactor, Butyrivibrio, Rothia, *Pseudomonas*, and Pediococcus**↑**; Actinobacteria, Millisia, Leifsonia, Paracoccus, and Eggerthella**↓**;	restoration of gut dysbiosis↑; BUA, inflammatory pathway and oxidative stress↓	Lin et al. (2020)
TCM(H)	Simiao decoction	Male C57BL/6 mice with GA	phylum Proteobacteria and genus *Helicobacter*	Pain threshold and repairing intestinal pathology↑, TNF-α, IL-6 and gut inflammation↓	Lin et al. (2020)
	Qu-Zhuo-Tong-Bi Decoction	C57BL/6 Mice with GA	butyrate-producing bacteria	Butyrate-producing bacteria, SCFAs and restored the intestinal barrier↑; inflammatory factors↓	Wen et al. (2020)
	Modified Baihu decoction	Male SD rats with AGA	Lachnospiraceae, Muribaculaceae, Bifidobacteriaceae, Lactobacillaceae, Erysipelotrichacea, Ruminococcaceae, Prevotellaceae and Enterobacteriaceae;	Restore the disturbance of the intestinal flora, BUA, IL-1β, NLRP3, ASC, and Caspase-1↓;	Wang et al. (2022a)
	Inulin	Uox-knockout C57BL/6J mice	Verrucomicrobia, *Bacteroides*, Parasutterella and Bifidobacterium↑; *Bacteroides*, Akkermansia and Ruminococcus↓;	Intestinal barrier function, and SCFA↑; BUA、LPS、inflamma-tory reaction↓	Guo et al. (2021)
	Chicory	quails	Bifidobacterium, Erysipelotrichaceae↑; Helicobacteraceae↓;	LPS, TLR4/NF-κB inflammatory pathway and BUA↓	Bian et al. (2020)
FMT(I)	the fecal microbiota from anserine-treated mice	diet-induced hyperuricemic mouse	Porphyromonas↓; *Lactobacillus* and *Clostridium*↑;	inflammation phenotypes, BUA, NLRP3 inflammasome, and and TLR4/MyD88/NF-κB signaling pathway ↓; uric acid excretion↑	Han et al. (2021a)
	Washed Microbiota Transplantation	patients with gout	not test	DAO,LPS, NLRP 3 and pro-IL-1β↓;	Xie et al. (2022)
Probiotics(J)	*Lactobacillus* brevis DM9218	HUA BALB/c male mice	Bacteroidetes↓ Proteobacteria, Akkermansia and Verrrucomicrobia↑	intestinal barrier integrity, BUA and IL-1β↓; XOD↑	An et al. (2014)
	*Lactobacillus* paracasei X11	HUA Balb/c mouse	*Lactobacillus*, Faecalibaculum, Muribaculaceae and Firmicutes↑; Bacteroidetes↓;	BUA, LPS, IL-1β,TNF-a↓; SCFAs and the antioxidant activity↑;	Cao et al. (2022)
	Limosilactobacillus fermentum JL-3 strain	HUA mice	Bacteroidetes↑; Firmicutes and Proteobacteria↓	NLRP3,IL-1β, MDA, CRE, BUN and BUA↓	Wu et al. (2021)

Note: DAO, diamine oxidase; BUA, blood uric acid; IL-6, interleukin-6; ASC, apoptosis-associated speck-like protein containing CARD; Caspase-1: cysteinyl aspartate specific proteinase-1; XOD, xanthine oxidase; BUN, blood urea nitrogen; CRE, creatinine.

Considering that most GA patients are treated with multiple drug combinations, the interactions between these therapeutic agents and the gut microbiome could be intricate *in vivo*. Moving forward, larger interventional clinical trials are warranted to explore the effects of specific pharmacological agents on the gut microbiota of GA patients.

### 5.2 Analgesic drugs from traditional Chinese medicine (TCM): effects on “gut-immune-joint” axis-mediated GA pain mechanisms

Clinicians frequently prescribe combinations of analgesic drugs with diverse pharmacological properties to enhance their analgesic effects (Szeto et al., 2020). However, such polypharmacy also elevates the risks of liver and kidney damage, cardiovascular events, and gastrointestinal reactions, in addition to the inherent toxicities of individual drugs. This significantly restricts the widespread clinical application of anti-gout medications. Consequently, GA remains challenging to treat, particularly the chronic form characterized by recurrent acute flares. Given its high efficacy and low toxicity, TCM is now considered a novel and promising analgesic therapy for GA (Han R. et al., 2021; Jiang et al., 2022). For example, *berberine* functions as a regulator of *p65Lys310* by inhibiting *p300* expression in macrophages, thereby exerting an anti-inflammatory effect in mice stimulated with acute LPS (Zhang C.-L. et al., 2021). Moreover, TCM can beneficially modulate cellular immune responses, inflammatory mediators, and chemokines.

TCM plays a pivotal regulatory role in cellular immune responses, inflammatory factors, and chemokines, with its influence extending through interactions with the gut microbiota (Xu et al., 2021) (Figure 2H). For instance, *berberine*, an isoquinoline alkaloid from the Chinese herbal medicine *Coptis chinensis Franch.* [Ranunculaceae; Coptidis rhizoma], has been shown to reduce joint inflammation and pain in GA mouse models (Xu et al., 2021). *Berberine* acts by inhibiting proteins like MyD88, TLR7 and NF-κB p65 through effects on the NLRP3/TLR signaling pathway (Zhang et al., 2023). It also downregulates the levels of inflammatory factors such as TNF-α and IL-6. Through these actions, *berberine* alleviates arthritis pain. In order to treat pain of GA, scholars discovered that *Qu-Zhuo-Tong-Bi decoction* might alter macrophage polarization and block the synthesis of pro-inflammatory factors by altering the prevalence of butyrate-producing bacteria and SCFA production in C57/Bl6J mice (Wen et al., 2020). Other herbal formulations, including *Si Miao San* (Lin et al., 2020), *Tongfu Ding* (Yang and Xing, 2021) and *Modified Baihu decoction* (Wang X. et al., 2022), as well as individual botanical drugs extracts, including *Cichorium intybus L.* [Asteraceae; Cichorii radix]*,* have all been demonstrated to be successful in preventing and treating GA pain (Bian et al., 2020; Han J. et al., 2021) (Table 1). Further exploration of the impact of botanical drug medications and formulations on the gut microbiota will contribute to a deeper understanding of how the gut microbiome and associated metabolites influence GA inflammation and pain.

### 5.3 Effect of fecal microbiota transplantation on GA pain

Fecal Microbiota Transplantation (FMT) stands as a contemporary and promising avenue of clinical research. This therapeutic strategy endeavors to reinstate gut microbial diversity by introducing microbiota from a healthy donor into the gastrointestinal tract of the recipient (Xie et al., 2022). FMT presents an innovative approach for addressing GA by modulating BUA levels, considering the intricate connection between BUA and gut microbial equilibrium (Figure 2I). While human research on FMT for health is still in its early stages, this technique emerges as a promising new modality for GA patients, meriting further investigation (Table 1). In a 14-month study, C57BL/6 male littermates were fed high-fat/high-cholesterol (HFHC), high-fat/low-cholesterol or normal chow. HFHC-fed mice received atorvastatin. Germ-free mice were transplanted with faces from mice fed different diets. Analysis included 16S rRNA sequencing for gut microbiota and LC-MS metabolomics for serum. As reported, germ-free mice gavages with HFHC-fed stools exhibited hepatic lipid accumulation, inflammation and cell proliferation (Zhang X. et al., 2021).

### 5.4 Effect of probiotics on GA pain

*Lactobacillus, Bifidobacterium,* and *yeast* have a longstanding history of application as probiotics in disease treatment (Zhao H. et al., 2022). Currently, probiotics exhibit promise for the treatment of GA (Zeng et al., 2022). Progress has been made in the utilization of probiotics for GA treatment. Probiotics seem to mitigate GA inflammation through two primary mechanisms (Figure 2J): inhibiting the inflammatory response and restoring the compromised intestinal epithelial barrier. For instance, one study investigated *Limosilactobacillus fermentum JL-3*, derived from “*Jiangshui*” (a Chinese fermented food). *In vitro*, *JL-3* demonstrated a high capacity to degrade UA. Oral administration to mice for 15 days resulted in sustained colonization of *JL-3* in the feces. Mice fed *JL-3* had urinary UA levels similar to the control but significantly lower serum UA levels (31.3%), confirming the UA-lowering effect of *JL-3. JL-3* also attenuated inflammatory and oxidative stress markers associated with hyperuricemia, and analysis of gut microbial diversity revealed its regulation of HUA-induced dysbiosis (Wu et al., 2021). In another experiment, mice underwent normal, high-fructose, or high-fructose with *DM9218* diets, analyzing metabolic parameters, fructose- and UA-related metabolites, and fecal microbiota. The *Lactobacillus* strain *DM9218* reduced serum UA and hepatic xanthine oxidase activity in fructose-fed mice. It mitigated high-fructose-induced intestinal dysbiosis by improving intestinal barrier function and reducing liver lipopolysaccharide. This aligned with downregulated inflammatory cytokine-stimulated xanthine oxidase expression and activity (Wang et al., 2019). In summary, probiotics enhance immune function, facilitate intestinal barrier repair, and regulate gut microbial balance to alleviate overall GA discomfort (Table 1).

## 6 Conclusion and outlook

In the realm of biological sciences, microbiomics has emerged as a critical and indispensable field of study (Sorbara and Pamer, 2022). Considerable research has explored the connection between gut microbes and arthritis diseases (Ramasamy et al., 2021; Wang et al., 2021). Further investigations are essential to comprehend the evolution of arthritis, its control of inflammation, and the restoration of the compromised microecological environment. Dysbiosis of the gut microbiota is recognized as a key factor influencing the onset and progression of several metabolic diseases. Robust evidence from both animal models and clinical studies establishes a compelling link between alterations in the gut microbiota during the development of GA and subsequent immune responses. Consequently, targeting the gut microbiota emerges as a promising preventive and therapeutic strategy for GA and HUA.

The therapeutic approach for GA involves nuanced modulation of gut microbiota types and abundance, alongside their metabolites, to regulate immune-inflammatory responses, particularly those mediated by macrophages. Nonetheless, the translation of gut microbiota-based therapies from animal models to clinical contexts encounters multifaceted challenges, encompassing concerns about the safety and stability of the gut microbiota, optimal intervention duration, patient adherence to treatment plans, and potential impacts on existing GA therapies. Anticipated future developments involve increased utilization of immunotherapeutic strategies for the prevention and treatment of GA, contributing to a more refined theoretical understanding and improved prognosis for patients with this condition. Moreover, to precisely elucidate the contributions of the gut microbiota to GA pain pathogenesis, forthcoming clinical investigations must be more rigorous and systematic (Song et al., 2023). This approach will enhance our comprehension of how the gut microbiome modulates inflammation and pain in individuals with GA.
